# Senescent cell accumulation mechanisms inferred from parabiosis

**DOI:** 10.1007/s11357-020-00286-x

**Published:** 2020-11-25

**Authors:** Omer Karin, Uri Alon

**Affiliations:** Department of Molecular Cell Biology, Weizmann Institute of Science, 76100 Rehovot, Israel

**Keywords:** Aging, Senescent cells, Parabiosis, Mathematical modeling, Systems biology

## Abstract

Senescent cells are growth-arrested cells that cause inflammation and play a causal role in aging. They accumulate with age, and preventing this accumulation delays age-related diseases. However, the mechanism for senescent cell accumulation is not fully understood. Accumulation can result from increasing production or decreasing removal of senescent cells with age, or both. To distinguish between these possibilities, we analyze data from parabiosis, the surgical conjoining of two mice so that they share circulation. Parabiosis between a young and old mouse, called heterochronic parabiosis, reduces senescent cell levels in the old mouse, while raising senescent cell levels in the young mouse. We show that parabiosis data can reject mechanisms for senescent cell accumulation in which only production rises with age or only removal decreases with age; both must vary with age. Since removal drops with age, senescent cell half-life rises with age. This matches a recent model for senescent cell accumulation developed from independent data on senescent cell dynamics, called the SR model, in which production rises linearly with age and senescent cells inhibit their own removal. The SR model further explains the timescales and mechanism of rejuvenation in parabiosis, based on transfer of spare removal capacity from the young mouse to the old. The present quantitative understanding can help design optimal treatments that remove senescent cells, by matching the time between treatments to the time it takes senescent cells to re-accumulate.

## Introduction

Senescent cells are growth-arrested cells that inhibit tissue regeneration and secrete pro-inflammatory factors called SASP (Senescence-Associated Secretory Phenotype) [1–3]. They have beneficial roles in development and wound healing. However, with age their abundance increases in multiple tissues [4–7], raising the concentration of pro-inflammatory SASP factors.

The accumulation of senescent cells plays a causal role in aging, as was demonstrated in mice studies. Continuous senescent cell ablation starting in midlife extended lifespan and delayed age-related disease [8], whereas senescent cell transplantation shortened lifespan and healthspan [9]. Senescent cell ablation in mice, using drugs or genetic means, improved tissue regeneration and delayed disease in a wide range of disease models including models for cardiovascular disease, diabetes, kidney failure, Alzheimer’s disease, and osteoarthritis [10–24].

Senescent cells have a lifetime on the order of days to weeks [25] and are therefore continually produced and removed over the lifespan. Their removal is carried out by immune cells including NK cells and macrophages [26].

The importance of senescent-cell accumulation for aging raises the question of how they accumulate with age. Understanding their accumulation mechanism can help to better design treatments to delay age-related diseases. In particular, it is unclear whether accumulation is due to an increase in production rate, a decline in removal rate, or both. We have recently addressed this question by modeling the dynamics of senescent cell accumulation [25]. The model, called the SR (saturating removal) model, was developed by comparing a class of mechanisms to data on senescent cell longitudinal dynamics [4] and to direct measurements of bleomycin-induced lung senescent cells in mice. This study concluded that senescent cell accumulation results from a combination of two effects. The first effect is a linear increase in senescent cell production rate with age. The second effect is a removal rate that is inhibited by the senescent cells themselves. The inhibition of removal can be due to factors secreted by senescent cells, or to saturation or exhaustion of the relevant immune cells by rising senescent cell numbers. The SR model was able to capture several features of senescent cells in mice, including mortality curves and the widening variation between individuals with age. It would be important to test the SR model with an independent set of experimental data.

Here, we independently test mechanisms for senescent-cell accumulation by considering a paradigm of aging research—*heterochronic parabiosis*, the surgical pairing of a young and old animal that conjoins their circulation. Parabiosis between equally aged animals, called isochronic parabiosis, is extensively used to study the effects of circulating factors such as hormones, metabolites, and immune cells on physiological function. The first study employing heterochronic parabiosis was performed by Ludwig and Elashoff, which showed that rat heterochronic parabionts lived 20% longer than control isochronic parabionts [27]. More recently, heterochronic parabiosis was demonstrated to stimulate the regeneration of multiple tissues in old mice [28–34] at only a slight expense to the young [35]. The mechanisms by which heterochronic parabiosis exerts its benefits remain unclear. Effects are attributed to blood-borne factors such as pro-inflammatory factors [30], or to the recruitment of young immune cells [31]. For example, immune cells from a young GFP-labeled mouse were recruited to repair neural lesions in the old parabiont [31].

An important step in understanding parabiosis was recently presented by Yousefzadeh et al. [36], who tested the effect of parabiosis on senescent cells. Yousefzadeh et al. quantified senescent cell gene expression markers and SASP markers in multiple tissues after isochronic and heterochronic parabiosis. They found that heterochronic parabiosis reduced senescent cell load in the old parabiont and mildly increased senescent cell load in the young parabiont. This suggests that the rejuvenating effects of parabiosis may be due, at least in part, to reduction of senescence cell accumulation in the old mouse.

Here, we use the data of Yousefzadeh et al. to test a wide class of mechanisms for senescent cell accumulation. For this purpose, we develop a mathematical model for senescent dynamics after parabiosis. We find that the data of Yousefzadeh et al. can reject many possible mechanisms, and in particular those with only rising production or only decreasing removal with age. The data indicates that the mechanism for senescent cell accumulation must include both an increase in production and a decrease in removal with age. The effects of heterochronic parabiosis on senescent cells are quantitatively consistent with the SR model developed in Karin et al. [25]. This provides a mechanism for parabiosis in which senescent cell removal capacity from the young mouse is transferred to the old. The model predicts an increase in lifespan of 20% in heterochronic parabionts compared with isochronic controls, similar to the experimental observations of Ludwig and Elashoff. The drop in removal rate with age means that senescent cell half-life rises with age, suggesting that optimal treatments to remove senescent cells can be given infrequently, potentially reducing side effects.

## Results

### Parabiosis data can reject several mechanisms for senescent cell accumulation

We begin with a formal analysis of possible mechanisms for the accumulation of senescent cells with age and show that many of them can be discarded based on the parabiosis data of Yousefzadeh et al.

**Fig. 1:**
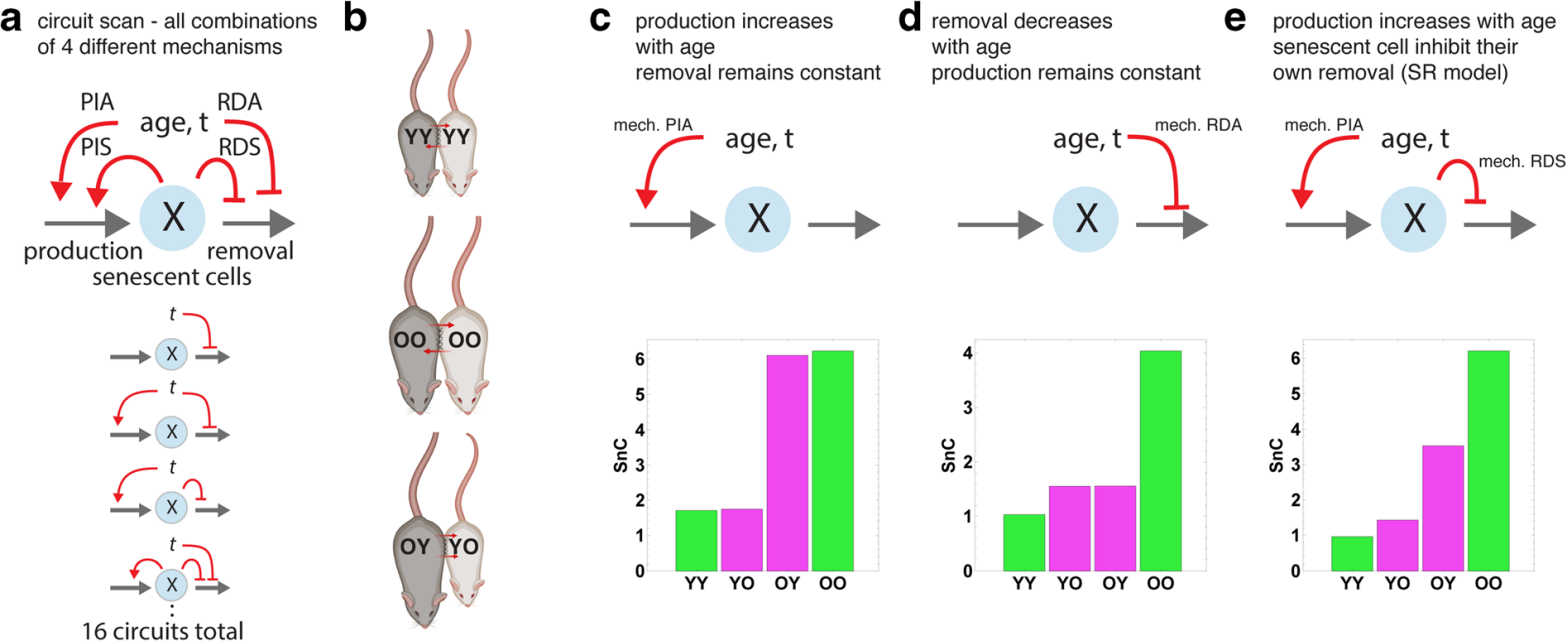
The effects of heterochronic parabiosis on senescent cells can be predicted from models of accumulation dynamics. **a** We modeled 16 mechanisms for the accumulation of senescent cells that include all combinations of increasing production with age (PIA), autocatalysis (PIS), senescent cell–independent decrease in removal with age (RDA), and senescent cell–dependent inhibition of removal (RDS). **b** Parabiosis is the surgical conjoining of mice to share circulation. The mice therefore share the immune-based removal mechanism for senescent cells. Isochronic (same-age) parabionts are labeled as YY (young) or OO (old). Heterochronic parabionts are labeled YO for the young parabiont and OY for the old parabionts. **c** A model with only an increase in production rate with age (PIA) does not show a change in senescent cell abundance after parabiosis. **d** A model with only an age-dependent decrease in clearance function (RDA), e.g. due to age-related impairment of immune cells, does not show a difference between the heterochronic young parabiont and the heterochronic old parabiont. **e** The SR model, which includes both an increase in production with age and an inhibition of senescent cells of their own removal process (PIA+RDS), shows an order of senescent cell abundance YY < YO < OY < OO

Accumulation is driven by a balance between the production and removal of senescent cells. The observed accumulation with age requires that either production increases, or removal decreases, or both. There are at least four possible mechanisms (Fig. 1a) [25]: (i) production increases with age (abbreviated PIA), (ii) autocatalysis, in which production increases with senescent cell concentration (abbreviated PIS), (iii) removal decreases with age in a senescent cell–independent manner (abbreviated RDA), and (iv) removal decreases with senescent cells (abbreviated RDS). The previously proposed SR model is a combination of PIA and RDS.

These four mechanisms can be combined in 16 possible ways. The 16 models make predictions for the relative order of senescent cells in parabiosis (Fig. 1b–e). Each model predicts a specific order for senescent cell load in YY (isochronic young), YO (young parabiont in heterochronic parabiosis), OY (old parabiont in heterochronic parabiosis), and OO (isochronic old). For example, a model with only production increase with age (PIA) predicts that YY=YO and that OY=OO (Fig. 1c). Importantly, the orders predicted by the models are not dependent on parameters.

We compared these predictions to the data of Yousefzadeh which tested how parabiosis affects senescent cell levels in mice [36]. Yousefzadeh et al. performed heterochronic and isochronic parabiosis between young (4 months old) and old (18 months old) mice, with *n* = 8 mice per group. They sacrificed the mice after 2 months of being conjoined and measured senescent cell markers (*p16*^*Ink4a*^ and *p21*^*Cip1*^) and SASP markers (Il1β, Il6, Mcp1, and Tnfα) in the heart, liver, kidney, lung, skeletal muscle, pancreas, and brain.

**Fig. 2:**
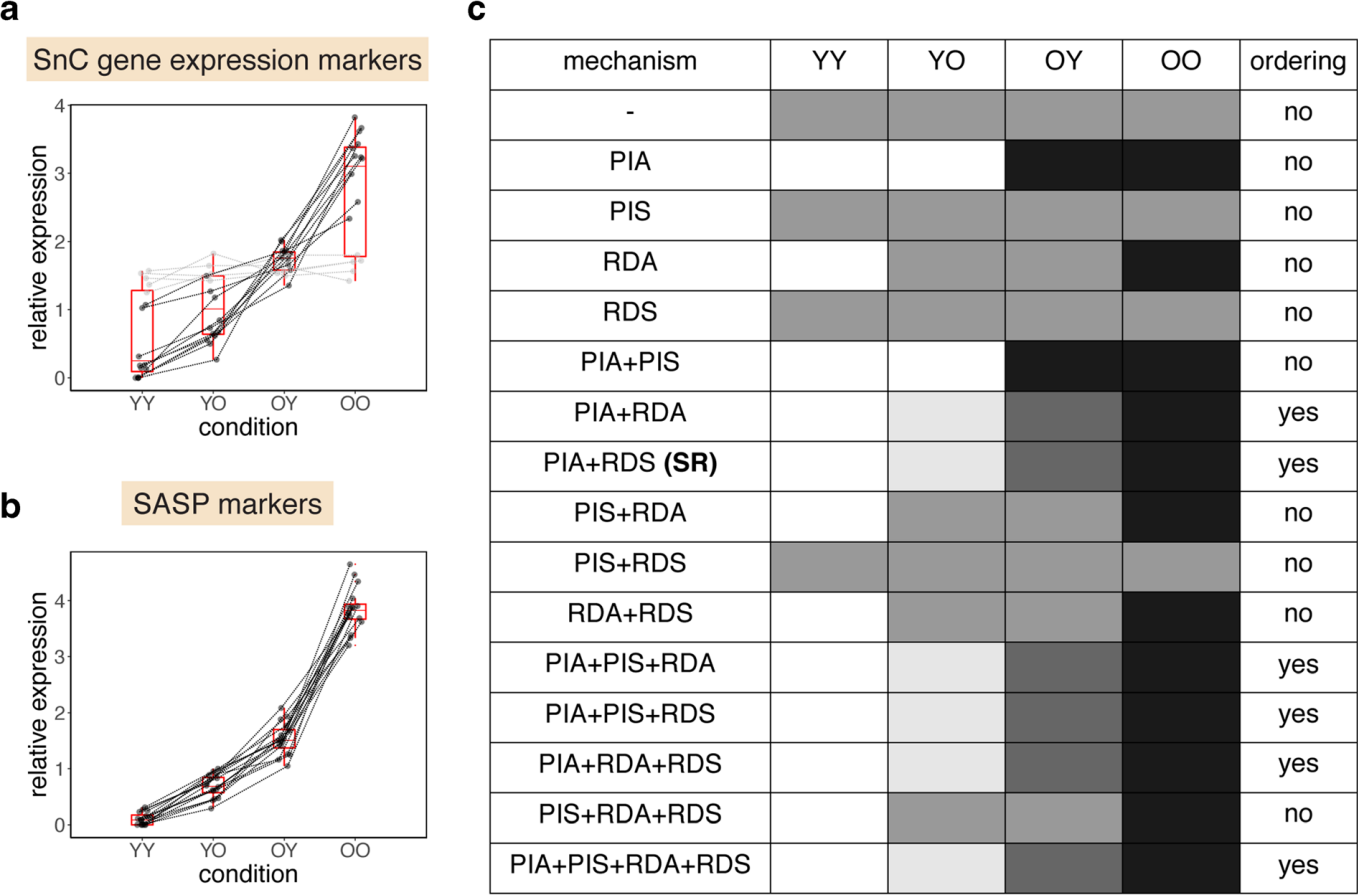
Order of senescent cell levels after parabiosis in old and young mice. A comparison of senescent cell abundance in young isochronic parabionts (YY), old isochronic parabionts (OO), young heterochronic parabionts (YO), and old heterochronic parabionts (OY). Senescent cell abundance was estimated by using gene expression markers (*p16*^*Ink4a*^ and *p21*^*Cip1*^) (**a**) in the liver, kidney, lung, pancreas, forebrain, cerebellum, gastrocnemius, and heart, and SASP markers (Il1β, Il6, Mcp1, and Tnfα) (**b**) in the liver, kidney, lung, and forebrain. The results of each test are presented as a line which connects the results for each of the parabiosis groups. Tests where there is a significant difference between YY and OO (and are therefore indicative of senescent cell accumulation) are shown in black. Data is from Yousefzadeh et al. [36]. To normalize for differences in average expression between markers and tissues, each line was normalized to have mean = 1. **c** Predictions for the ordering of senescent cell levels for YY, YO, OY, and OO for all 16 mechanisms described in Fig. 1 (darker color corresponds to higher senescent cell levels). Only models where both production increases with age (mechanism PIA) and removal decreases with age or senescent cells (mechanisms RDA or RDS) result in YY < YO < OY < OO as observed

We reanalyzed this data, by normalizing the mean of each marker in each tissue to one, which allows easy comparison of the ordering of the different parabiont mice in terms of marker intensity. Strikingly, senescent cell levels show a consistent order between the groups (Fig. 2). Young isochronic parabionts (YY) have lower senescent cell levels than young heterochronic parabionts (YO), which have lower senescent cells than old heterochronic parabionts (OY), which, finally, have lower senescent cells than old isochronic parabionts (OO). This holds for all tissues where there is a significant difference in the marker expression level between the young and old isochronic groups (Fig. 2a, *p* = 9·10^−4^ Wilcoxon signed-rank test for each ordering). It also holds for all SASP markers in all tissues (Fig. 2b, *p* = 6·10^−5^ for Wilcoxon signed-rank test for each ordering). Thus, parabiosis systematically modulates senescent cell levels with the order YY < YO < OY < OO.

We find that 10 of the 16 models cannot explain this order (Fig. 2c). Their inability is not dependent on specific parameter choices but is rather built into the logic of the models. We next explain this intuitively and provide more details in the “Materials and methods” section.

We begin with models in which senescent cell accumulation is due only to increasing production with age, whereas the removal rate is constant (age- and senescent cell–independent). These models are mechanism PIA or PIA + PIS, in Figs. 1 and 2c. If the rise in production with age is due to systemic circulating factors, these factors are mixed between the mice after parabiosis. Both parabionts therefore have the same production rate, and, as assumed in the model, also the same removal rate, and thus OY=YO, in contradiction to the observed lower senescent cell load in the younger parabiont, YO < OY.

If the rise in production with age is due to local tissue factors and is not mixed in the circulation, production rate in the old parabiont would be higher than in the young one. The removal rate is assumed to be age-independent and is thus the same in young and old mice. The model therefore predicts that heterochronic and isochronic parabiosis have the same effects: OO=OY and YY=YO, as opposed to the data YY < YO and OY < OO. These considerations discount mechanisms in which accumulation is only due to rising production.

Similarly, models in which accumulation is due only to a drop in removal rate with age (mechanism RDA) cannot explain the observed difference between YO and OY. Both the young and old heterochronic parabionts should have identical removal rates because the immune cells that remove senescent cells, together with all circulating factors, are mixed between the mice. They also have the same production rates as assumed in this model variant. Thus, the parabionts should have the same amount of senescent cells, YO=OY. This prediction does not describe the observed lower senescent cell levels in the younger parabiont, YO < OY. We note that a model with only mechanism RDS is also inappropriate because senescent cells do not accumulate with age at all.

In the present class of models, only models that have both an increase in production with age (mechanism PIA), as well as a drop in removal rate, due to either mechanism RDA or RDS, can explain the observed changes in senescent cells after parabiosis. Mechanism PIS does not affect the model conclusions qualitatively. Viewed in this way, the experiment of Yousefzadeh et al. helps to restrict the range of senescent cell accumulation dynamical models.

The parabiosis data can also make quantitative predictions about the relative increase in production rate and decrease in removal rate with age (“Materials and methods” section). This prediction is based on the fact that steady-state senescent cell levels are the ratio of their production rate and per-cell removal rates.

Removal is predicted to drop by a factor of (YY·OY)/(OO·YO) = 0.16 ± 0.06 based on gene expression markers and a factor of (YY·OY)/(OO·YO) = 0.06 ± 0.01 based on SASP markers (error bars from paired bootstrapping). Thus, the removal rate falls by a factor of 5–20, and hence, senescent cell lifetime rises by the same factor. This is in reasonable agreement with direct measurements of lung Bleomycin-induced senescent cell removal in young and old mice [25], in which senescent cell half-life rose by a factor of about 5 (5 ± 1 days versus 25 ± 6 days).

### SR model can explain the effect of parabiosis on senescent cells

The SR model belongs to the class of models that can explain the parabiosis experiments, despite the fact that the SR model was developed based on an independent set of experiments on senescent cell dynamics in a non-parabiosis setting. The SR model has mechanisms PIA + RDS and predicts YY < YO < OY < OO as observed.

In the “Materials and methods” section, we show the full derivation of the SR model for parabiosis, denoted the parabiosis-SR model. Here, we describe the important intuitive aspects of this derivation. The parabiosis-SR model posits that senescent cells are in rapid turnover relative to the lifespan, that senescent cell production increases linearly with age, and that removal is inhibited or saturated by senescent cells [25]. At young ages, efficient removal keeps senescent cells low. At old ages, increased production leads to high senescent cell loads that inhibit their own removal, leading to even higher load. Senescent cells accumulate at an accelerating pace with age.

To model parabiosis, we note that after parabiosis the two mice share circulation, but the senescent cells in each mouse stay within its tissues. As a result, the blood-borne removal mechanism (immune cells) is shared by the two mice. The immune cells see the senescent cells and secreted factors in both mice and are therefore inhibited by the averaged senescent cell concentration of the two mice. The production of senescent cells in each mouse and the noise terms are assumed to be unaffected by parabiosis.

The parabiosis-SR model makes predictions for parabiosis that do not depend on the model parameters. Since removal is inhibited by the *averaged* senescent cell concentrations in the two mice, the removal rate of the old parabiont is increased compared to isochronic old mice. The intuitive reason is that the young mouse has spare removal capacity, part of which is transferred to the old mouse by the shared circulation. As a result, senescent cell load in OO is greater than in OY, because they have equal (age-dependent) production but the latter has greater removal rate. Thus, OY < OO.

For the same reason, the averaging of inhibition decreases removal rate in the young parabiont compared to isochronic young mice. The spare removal capacity of the young mouse is transferred to the old mouse, slowing removal in the young heterochronic parabiont. As a result, senescent cell load in YY is lower than in YO, YY < YO. Finally, since the production rate in the old mouse is greater than that in the young mouse, and removal rate is the same in the two parabionts due to the shared circulation, a heterochronic pair shows YO < OY.

The SR model thus predicts a strict order of senescent cell levels YY < YO < OY < OO, without dependence on model parameters (see “Materials and methods” section for further details). This matches the experimental observation of Fig. 2.

The model has additional quantitative predictions. The parabiosis-SR model posits a specific way that production rises with age (mechanism PIA), namely a linear increase with age. This predicts that the ratio of senescent cells in the parabionts (OY/YO) is the ratio of their production rates, namely the ratio of their ages, which in the present case is 20 months/6 months ~ 3.3 (assuming that mechanism PIS is absent or small). A more precise calculation using the full stochastic SR model equations with the paraemters of Ref [25] shows that the ratio is about 2.5. This is in agreement with the observed ratios. The production rate between ages 6 and 20 months is estimated to rise by a factor of OY/YO = 2.9 ± 0.6 based on gene expression markers and OY/YO = 2.4 ± 0.2 based on SASP markers (error bars from paired bootstrapping).

**Fig. 3:**
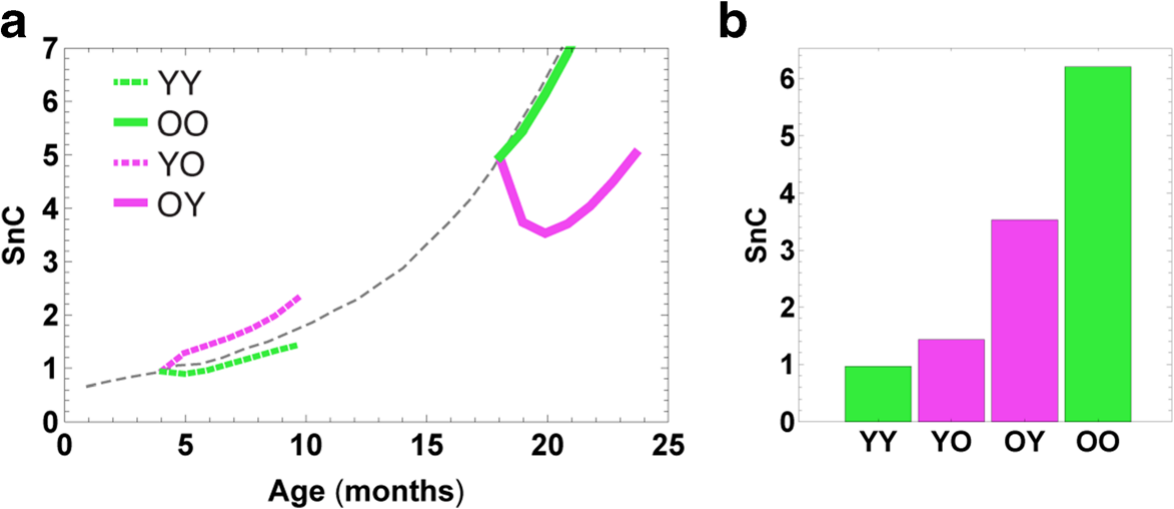
SR model provides an explanation for the change in senescent cell levels after parabiosis. **a** The SR model, parameterized according to Karin et al. [25], was simulated before and after parabiosis, using Eq. 3. After isochronic parabiosis, there is a slight decrease in senescent cells in both young (YY, dashed green line) and old (OO, solid green line) parabionts, compared with baseline (dashed gray line). On the other hand, there is a large decrease in senescent cells in old heterochronic parabionts (OY, solid purple line), and a slight increase in the young heterochronic parabionts (YO, dashed purple line). The changes occur on the timescale of a few weeks due to senescent cell turnover. **b** After 2 months of parabiosis, there is a clear order of senescent cell abundance YY < YO < OY < OO, similar to the effect shown in Fig. 2. Model parameters are provided in the “Materials and methods” section

In addition to these considerations, the model can predict the temporal dynamics of senescent cells following parabiosis. To address this, we simulated the effect of parabiosis on senescent cell levels using stochastic simulations of the parabiosis-SR model (Fig. 3). We used the parameters inferred for senescent cell dynamics in mice from Karin et al. [25] and set the age of the young and old parabionts at 4 months and 18 months at the time of conjoining, as in the experiments of Yousefzadeh et al. [36]. Figure 3a shows the simulation results for the dynamics of senescent cells .Within weeks, heterochronic parabiosis results in a large drop in senescent cells in the old mouse compared with isochronic parabiosis (OY compared to OO, Fig. 3a, b). For young mice, on the other hand, heterochronic parabiosis results in a slight elevation of senescent cells compared with the isochronic case (YO compared to YY, Fig. 3a, b). This agrees with the results of Yousefzadeh et al. [36] presented in Fig. 2.

The simulations indicate the timescale of the changes in senescent cells after parabiosis. The drop in senescent cells in OY is seen within about a month, which corresponds to the lifetime of senescent cells in old mice, experimentally determined in Karin 2019.

#### SR model can explain the effect of parabiosis on lifespan

**Fig. 4:**
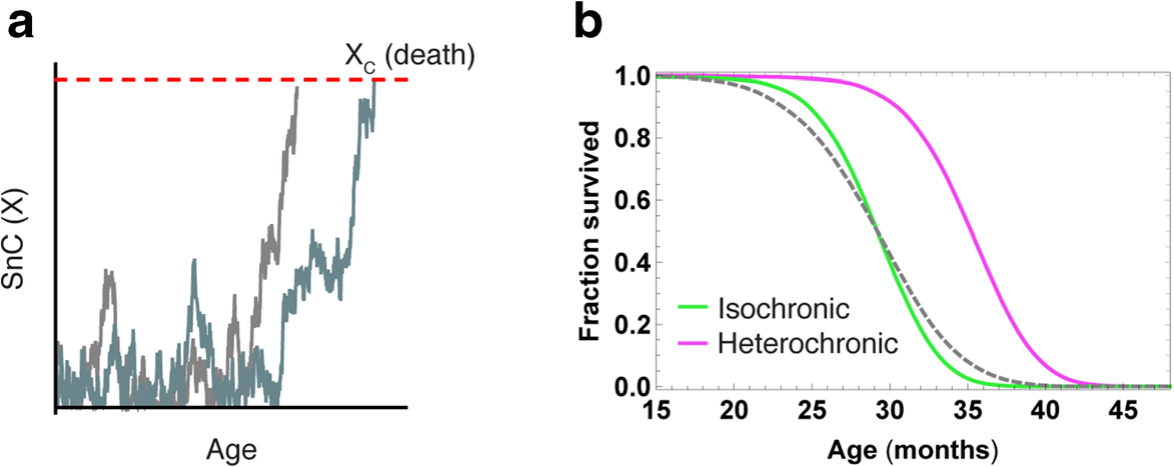
SR model predicts an increase in lifespan after heterochronic parabiosis compared with isochronic parabiosis. **a** To model the effects of the reduction in senescent cell load on lifespan, we used the simple assumption taken in Karin et al. [25] that death occurs when senescent cells exceed a critical threshold *X*_C_ (in this case, *X*_C_ = 17). **b** Heterochronic parabiosis (solid purple) increases the mean lifespan by 20% compared with isochronic parabiosis (solid green). Baseline is marked by a dashed gray line

We next use the parabiosis-SR model to test the effect of parabiosis on lifespan. To model lifespan, we add the assumption that death occurs when senescent cells cross a critical threshold [25] (Fig. 4a). We used the threshold value and SR parameters of Karin et al. which were found to describe mouse survival curves well [25]. We find that heterochronic parabiosis results in a lifespan extension of approximately 20% compared with isochronic parabiosis in the model (Fig. 4b). This is similar to the lifespan extension reported by Ludwig and Elashoff [27].

#### New experiments can test additional aspects of senescence in parabiosis

**Fig. 5:**
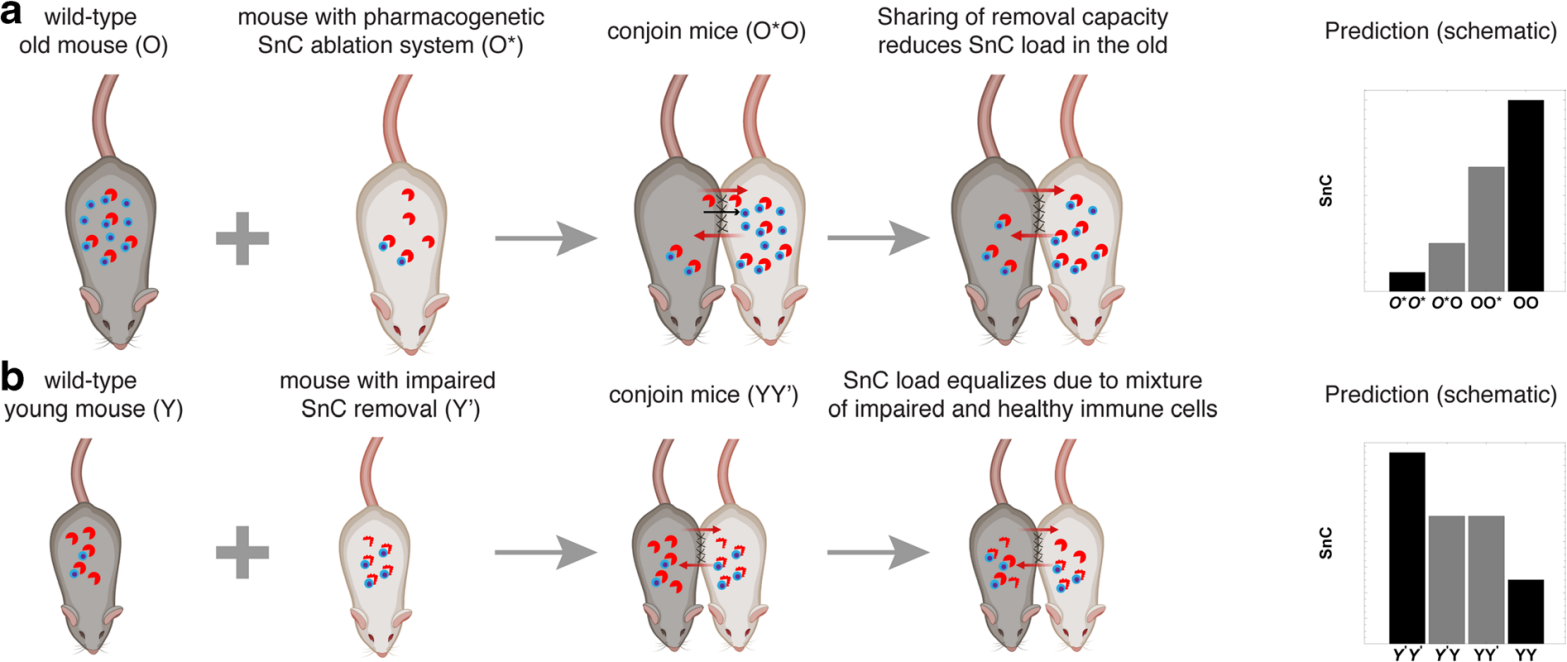
Suggestions for experiments to test mechanisms for senescent cell dynamics. **a** To test whether senescent cells indeed inhibit their own removal, we suggest the following experiment. A wild-type old mouse (O) may be conjoined with a mouse that has a pharmacogenetic construct that allows for continuous drug-induced removal of senescent cells, like the strain described in [8], which we denote (O*). This causes the removal of senescent cells only in one of the two parabionts. If this reduces senescent cells in the other parabiont as well, this suggests that senescent cells indeed inhibit their own removal. **b** To test for the effects of impaired senescent cell removal after parabiosis, we suggest that young wild-type mice (Y) may be conjoined with mice with impaired senescent cell removal, such as the perforin knock-out mice described in [26], which we denote Y′. These mice have higher senescent cell levels. After parabiosis, their senescent cells are predicted to reduce and be the same as their conjoined wild-type parabiont

The present framework suggests new experiments to gain further insight. The data of Yousefzadeh et al. is consistent both with a senescent cell–dependent drop in removal (as in the SR model, mechanism RDS), and with an intrinsic age-dependent drop in senescent cell removal rate that is not senescent cell–dependent (mechanism RDA). To distinguish between these mechanisms, consider the following experiment. Parabiosis is performed between two old mice, one of which has a pharmacogenetic system which allows ablating senescent cells upon the addition of a drug [8]. The model predicts that ablation in one parabiont will affect senescent cell levels in the other parabiont. This tests the SR model feature that senescent cells inhibit their own removal (Fig. 5a). If senescent cells in the other parabiont remain unaffected, the experiment would support the conclusion that senescent cells do not affect their own systemic removal rate.

A second informative experiment is isochronic parabiosis between a wild-type mouse and a mouse with defective senescent cell clearance. Defective clearance was established, for example, in perforin-deficient mice [26]. Perforin deficiency is, in a sense, equivalent to mechanism RDA, and because the mice are isochronic, they should have the same senescent cell production rate. In this case, the model predicts that the mutant mice will have higher senescent cells before parabiosis, but that a month or more after parabiosis, both the mutant and the wild-type parabionts will have similar senescent cell levels (Fig. 5b).

### The SR model can help to design optimal senolytic treatment

Recent studies show that senolytic drugs or immune therapy approaches can remove senescent cells in mice and humans [9, 22, 37]. Since these treatments are likely to have side effects, it is important to design intermittent therapy with the largest possible time interval between treatments [38]. An important consideration is the time that it takes senescent cells to re-accumulate after they are removed. To calculate this re-accumulation time requires a precise understanding of the production and removal rates of senescent cells.

**Fig. 6:**
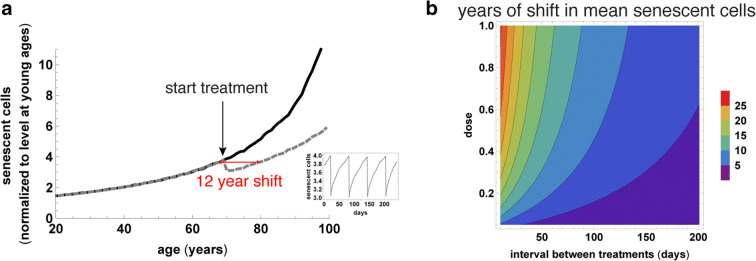
The SR model suggests that senolytic treatment starting at old age can have a large effect even if given infrequently. **a** Average senescent cell abundance trajectories with recurrent treatment with senolytics (gray dashed) and without (black). The senolytic is applied once every 60 days, starting from age 70 years, and each dose removes about half of the senescent cells present. The recurrent treatment with senolytics reduces senescent cell levels, which then re-accumulate until the next treatment (*Inset*). This re-accumulation takes longer the longer the senescent cell half-life. **b** The effectiveness of senolytic treatment starting at age 70 as a function of senolytic dose (fraction of senescent cells killed) and interval between treatments. Effectiveness is measured as number of years in which the senescent load at age 80 is shifted to levels typical at younger ages due to the treatment. Trajectories were simulated using the SR model with human parameters (see “Materials and methods” section), assuming that senolytics are effective against a susceptible subpopulation of senescent cells which consists of 25% of the senescent cells.

We therefore use the SR model with parameters calibrated for humans in Ref [25] to simulate senescent cell removal therapy with different temporal spacing between treatments. We find that treatment can be as infrequent as once per 2 months in order to maintain average senescent cell levels that are about 2-fold lower than without therapy (Fig. 6a, b). This amounts to a shift to senescent cell levels typical of a person 12 years younger. Thus, intermittent treatment with senolytics or immune therapy starting at old age and given once every 2 months may be a reasonable approach in humans.

## Discussion

Senescent cells drive aging and aging-related pathologies, and it is therefore important to understand how they accumulate with age. We scanned a class of senescent cell accumulation mechanisms and find that parabiosis data can reject many of these mechanisms. Among the rejected mechanisms is accumulation driven only by a rise in senescent production with age, or only by reduction in senescent cell removal with age. The parabiosis data indicates that both productions must rise with age and removal must drop with age. This aligns with the SR model which was developed based on independent data on senescent cell dynamics in a non-parabiosis setting. The SR model posits that senescent cells inhibit their own removal. It further predicts the timescale of accumulation in parabiosis and explains the effect of parabiosis on lifespan. Thus, parabiosis, together with previous work on senescent cell dynamics, offers a compelling model for senescent cell accumulation with age.

The SR model predicts that parabiosis strongly reduces senescent cell load in the old parabiont and raises the load slightly in the young parabiont. This quantitatively matches the experiments of Yousefzadeh et al. on senescent cell levels after parabiosis across many tissues and multiple markers. The model offers the following intuitive explanation for rejuvenation of the old parabiont: parabiosis causes a mixing of removal capacity, allowing the spare removal capacity of the young mouse to enhance removal in the old mouse.

The observation that parabiosis affects senescent cell levels in all measured tissues in the same direction and at approximately the same relative magnitudes suggests a systemic control of senescent cell abundance. The SR model, with its sharing of removal among all tissues, predicts a coordination of senescent cell levels between tissues. This is due to the limited total removal capacity. An increase in senescent cells in one tissue slows senescent cell removal from all other tissues, so their senescent cell levels tend to rise as well. This coordinates senescent cell levels across tissues, thus potentially helping to synchronize aging phenotypes across organs. Although individuals at old age differ vastly from each other in physiological performance, the SR model predicts relatively similar physiological performance between tissues of a given individual [39–41].

An open question is whether senescent cell–mediated inhibition of removal is due to the saturation of immune cells or to inhibitory blood-borne factors associated with senescent cells, or both. This question can be addressed in principle by performing plasma exchange heterochronically and isochronically for several weeks and then measuring the effect on senescent cells. If plasma exchange is sufficient for reducing senescent cells, it would suggest that inhibition is due to blood-borne factors. Importantly, plasma exchange for only a day or a few days is not expected to be sufficient due to the estimated lifetime of senescent cells in old mice, on the order of one month.

The ratio of senescent cell levels in young and old parabionts allows an estimate of the relative increase in production with age. This estimate is consistent with the SR model that production rises linearly with age. One physiological origin for a linear rise with age is that in the aging process, senescent cells originate from stem cells that have acquired mutations that are silent in the stem cell, but that cause damage in their differentiated progeny cells. These damaged differentiated cells become senescent. Since stem cells divide at an approximately constant rate throughout adulthood, the number of such mutant stem cells should rise approximately linearly with age. This generates production of senescent cells at a rate that rises linearly with age.

The rejuvenation effects of parabiosis and of senescent-cell ablation span many tissues and markers. This suggests that intermittent senescent cell ablation, by senolytic drugs [38] or immune therapy [37], may be a promising direction for the Geroscience hypothesis [42, 43] that an intervention that retards the aging process will simultaneously delay the onset and severity of multiple age-related diseases. The present estimates for accumulation rates suggest that intermittent treatment at a frequency of 1–2 months may be sufficient to prevent accumulation and reduce senescent cell load in future treatment regiments.

## Materials and methods

### Statistical analysis

To test the order between senescent cell levels in the different conditions (YY < YO < OY < OO), we performed a Wilcoxon signed-rank test which compares matched samples, which in this case are tests for senescent cell levels (such as p21-lung) for different experimental conditions. This yields a significance level of *p* = 9·10^−4^ for the gene expression markers, and *p* = 6·10^−5^ for the SASP markers, for all the order comparisons. To account for the possibility that different markers for the same tissue are not independent, we considered the extreme possibility that the tissues are perfectly dependent and variation is due only to experimental noise. In this case, the signed-rank test for tissue averages yields a significance level of *p* = 0.014 for the gene expression markers and *p* = 0.046 for the SASP markers (the change in *p* is only due to the smaller sample size). Finally, an alternative analysis approach compares overall organism senescent cells by bootstrapping and averaging all tissues from the empirical distributions. This analysis yields *p* < 10^−3^ for all orderings.

### Circuit scan

We use the approach of Karin et al. [25] to analyze all combinations of four possible mechanisms for the accumulation of senescent cells. (i) increasing production rate with age (production increases with age, PIA), (ii) autocatalysis of production by paracrine effects (production increases with senescent cells, PIS), (iii) age-related decline in removal due to decline in immune surveillance that is senescent cell-independent (removal decreases with age, RDA), and (iv) senescent cells inhibit their own removal (removal decreases with senescent cells, RDS). The model for a single mouse that includes all four processes is:

1 $$ \dot{X}=p\left(X,t\right)-r\left(X,t\right)X+\sqrt{2\epsilon }{\xi}_t $$

where *t* is the age of the organism, *X* is senescent cell abundance, *p* is the senescent cell production rate and *r* is the per-cell removal rate of senescent cells. Mechanism PIA is given by *p*(*t*) which increases with age *t*, mechanism PIS by *p*(*X*) which increases with *X*, mechanism RDA by *r*(*t*) that decreases with *t* and mechanism RDS by *r*(*X*) that decreases with *X*. Senescent cell steady-state abundance results from a balance between production and removal ($\frac{dX}{dt}=0$), yielding:

$$ {X}_{ST}=\frac{p\left({X}_{ST},t\right)}{r\left({X}_{ST},t\right)} $$

We asked which mechanism combinations provide the order observed in senescent cell abundance after parabiosis (YY < YO < OY < OO). To address this, we derive a general equation for senescent cell dynamics after parabiosis from Eq. 1. Consider two mice with blood volumes *V*_1_, *V*_2_ (older mice are usually larger than younger mice). The concentration of senescent cells in each mouse is *X*_*i*_, with *i = 1*,*2*, and the senescent cells stay within the mouse tissues (we ignore here senescent circulating cells). After parabiosis removal becomes shared. The blood-borne removal mechanism (the immune cells) in the conjoined mice thus sees an averaged senescent cell concentration of $\overline{X}=\frac{V_1{X}_1+{V}_2{X}_2}{V_{tot}}$ here V_tot_=V_1_+V_2_. The removal rate results from averaging the removal rates of the mice following the mixing of immune cells due to shared circulation, inhibited by the average senescent cell concentration$\overline{X}$. Therefore:

2 $$ \dot{X_i}=p\left({X}_i,{t}_i\right)-\frac{1}{V_{tot}}\left({V}_1\ r\left(\ \overline{X},{t}_1\right)+{V}_2r\left(\ \overline{X},{t}_2\right)\right){X}_i+\sqrt{2\epsilon }{\xi}_t $$

Thus, after parabiosis, the steady-state senescent cell concentration in each mouse is:

3 $$ {X_i}_{ST}=\frac{p\left({X_i}_{ST},{t}_i\right)}{\frac{1}{V_{tot}}\left({V}_1\ r\left(\ \overline{X},{t}_1\right)+{V}_2r\left(\ \overline{X},{t}_2\right)\right)} $$

For isochronic parabionts, because *t*_1_ = *t*_2_, *V*_1_ = *V*_2_, we get *X*_1*ST*_ = *X*_2*ST*_, so that:

$$ {X_i}_{ST}=\frac{p\left({X_i}_{ST},{t}_i\right)}{r\left({X_i}_{ST},{t}_i\right)} $$

The steady-state of senescent cells in the isochronic case is therefore same as that before parabiosis.

In the heterochronic case, we asked which mechanism combinations provide the observed order YY < YO < OY < OO. We note that since removal is shared, the fact that YO < OY means, from Eq. 3, that production must rise with age (mechanism PIA).

Furthermore, removal must also decline. To see this, consider the case where removal is constant, in the sense that it does not depend on age or on senescent cells (no mechanism RDA or RDS). In this case, the denominator of Eq. 3 is the same in isochronic and heterochronic parabiosis. This means that heterochronic parabiosis does not affect *X* relative to the isochronic case: YY=YO and OY=OO. This is in contrast to the observed order YY < YO < OY < OO. We conclude that removal must decrease in the older mouse (mechanism RDA or mechanism RDS) and that production must rise with age (mechanism PIA).

We next show that mechanism PIA + RDA or PIA + RDS provide the observed order. Let *i* = 1 denotes the young parabiont and *i* = 2 denotes the old parabiont. Since the denominator in Eq. 3 is the same for both mice, and the numerator is smaller for the young mouse, YO < OY. Removal is slower for OO than for OY and faster for YY compared with YO, due to the effect of the other mouse on$\overline{X},$which results in YO > YY and OY < OO.

Thus, the combination of mechanism PIA and either mechanism RDA or RDS is the minimal property, within the present class, that ensures the observed effects of parabiosis on senescent cells. The results for all 16 mechanisms are shown in Fig. 2c. Notably, the order does not depend on parameters.

The present approach also allows estimating the change in production and removal rates with age, in the case where the production rate does not depend on *X*. The production rate in young and old mice is $\frac{p\left({t}_2\right)}{p\left({t}_1\right)}=\frac{OY}{YO}$, since the denominator in Eq. 3 is the same for the parabionts. In the case where production rates depend on *X*, this ratio may underestimate the actual changes in production with age, as can be seen when linearizing the production rates: $\frac{OY}{YO}=\frac{p\left({X}_{OY},{t}_2\right)}{p\left({X}_{YO},{t}_1\right)}\approx \frac{p\left(0,{t}_2\right)+\frac{\partial p}{\partial X}\left(0,{t}_2\right){X}_{OY}}{p\left(0,{t}_1\right)+\frac{\partial p}{\partial X}\left(0,{t}_1\right){X}_{YO}}\le \frac{p\left(0,{t}_2\right)+\frac{\partial p}{\partial X}\left(0,{t}_2\right){X}_{OO}}{p\left(0,{t}_1\right)+\frac{\partial p}{\partial X}\left(0,{t}_1\right){X}_{YY}}\approx \frac{p\left({X}_{OO},{t}_2\right)}{p\left({X}_{YY},{t}_1\right)}$. The removal rate ratio can be estimated from $\frac{YY}{OO}\cdotp \frac{OY}{YO}=\frac{p\left({X}_{YY},{t}_1\right)}{r\left({X}_{YY},{t}_1\right)}\cdotp \frac{r\left({X}_{OO},{t}_2\right)}{p\left({X}_{OO},{t}_2\right)}\cdotp \frac{p\left({X}_{OY},{t}_2\right)}{r_{joint}}\cdotp \frac{r_{joint}}{p\left({X}_{YO},{t}_1\right)}=\frac{p\left({X}_{YY},{t}_1\right)}{p\left({X}_{YO},{t}_1\right)}\cdotp \frac{p\left({X}_{OY},{t}_2\right)}{p\left({X}_{OO},{t}_2\right)}\cdotp \frac{r\left({X}_{OO},{t}_2\right)}{r\left({X}_{YY},{t}_1\right)}$. Again for the case where production is independent on *X*, we get $\frac{YY}{OO}\cdotp \frac{OY}{YO}=\frac{r\left({X}_{OO},{t}_2\right)}{r\left({X}_{YY},{t}_1\right)}$. More generally, in the case where production rates depend on *X*, since $\frac{p\left({X}_{YY},{t}_1\right)}{p\left({X}_{YO},{t}_1\right)}\cdotp \frac{p\left({X}_{OY},{t}_2\right)}{p\left({X}_{OO},{t}_2\right)}\le 1$, this is an underestimate for the ratio of the removal rates: $\frac{YY}{OO}\cdotp \frac{OY}{YO}\le \frac{r\left({X}_{OO},{t}_2\right)}{r\left({X}_{YY},{t}_1\right)}$.

### SR-parabiosis model

The SR model [25] posits a linearly increasing production with age and removal inhibited by senescent cells according to a Michaelis-Menten-like function:

4 $$ \dot{X_i}=\eta {t}_i-\frac{\beta {X}_i}{X_i+\kappa }+\sqrt{2\epsilon }{\xi}_t $$

where *t*_*i*_ is the age of organism *i*, *X*_*i*_ is senescent cell abundance, *η* is the rate of increase in production rate with age, *β* is the removal parameter, *κ* is the halfway saturation point of removal inhibition by senescent cells, and *ϵ* is the noise amplitude. The mice reference parameters are: *η* = 0.15 day^−1^year^−1^, *β* = 0.27 day^−1^, *κ* = 1.1, *ϵ* = 0.14 day^−1^ [25].

The parabiosis-SR equation for the senescent cell dynamics of each mouse has removal inhibition due to the average senescent cell concentration of the two mice:

5 $$ \dot{X_i}=\eta {t}_i-\frac{\beta }{\overline{X}+\kappa }{X}_i+\sqrt{2\epsilon }{\xi}_t $$

Solving Eq. (2) at steady-state gives the following quasi-steady-state for each mouse:

6 $$ {X_i}_{ST}=\frac{\kappa {V}_{tot}{\upeta \mathrm{t}}_i}{V_1\left(\beta -{\upeta \mathrm{t}}_1\right)+{V}_2\left(\beta -{\upeta \mathrm{t}}_2\right)} $$

The denominator is the same for both mice, but the numerator is proportional to the mouse age t_*i*_. Thus, for heterochronic parabiosis, we get YO < OY, and the ratio of senescent cell abundances goes as the ratio of ages. For the isochronic case, the mice have equal ages and volumes: *t*_1_ = *t*_2_ = *t* and *V*_*1*_ = *V*_2_ = *V*:

$$ {X_{ST}}_{Iso}=\frac{\kappa \upeta \mathrm{t}}{\beta -\upeta \mathrm{t}} $$

This is the same as without parabiosis. Comparing this to the aged-matched heterochronic case, we get:

$$ \frac{X_{ST}}{{X_{ST}}_{Iso}}=\frac{V_{tot}}{V_1+{V}_2\frac{1-{\mathrm{t}}^{\prime }/{t}_0}{1-\mathrm{t}/{t}_0}} $$

where *t*’ is the age of the other mouse, and ${t}_0=\frac{\beta }{\eta}\approx 22\ \mathrm{months}$ using the mouse reference parameter set. Note that this equation is only valid at ages *t*^′^ < *t*_0_, a condition which applies to the experiments of Yousefzadeh et al. Beyond *t*_0_, stochastic simulations of the SR model are needed to obtain accurate results. Thus, *X*_*ST*_ < *X*_*STIso*_ when $\frac{1-{\mathrm{t}}^{\prime }/{t}_0}{1-\mathrm{t}/{t}_0}>1$, which is equivalent to t > t′, so, *X*_*ST*_ < *X*_*STIso*_ for the older parabiont: OY < OO. For the same reason, *X*_*ST*_ > *X*_*STIso*_ for the young parabiont. The SR model therefore provides the order YY < YO < OY < OO. This order does not depend on parameter values.

### Mouse mortality

We model mortality as in [25], by assuming that death occurs when senescent cell levels *X* cross a critical threshold *X*_C_. The parameters used in Fig. 4 are the same as those used to model mouse mortality curves in [25]: *η* = 0.084 day^−1^year^−1^, *β* = 0.15 day^−1^, *κ* = 0.5, *ϵ* = 0.16 day^−1^, *X*_C_ = 17. For parabionts, we assume that death occurs when the first mouse dies (i.e. senescent cells cross the critical threshold).

### Senolytic drug simulations

In order to simulate the effect of senolytic drugs, we modified the SR model to include a drug-dependent removal term $-{\delta}_{t_0}\left(t,\lambda, \phi \right)X$ where ${\delta}_{t_0}\left(t,\lambda, \phi \right)$ is a function that represents the periodic application of a senolytic drug with dosage *λ* every *ϕ* days, starting from age *t*_0_:

$$ {\delta}_{t_0}\left(t,\lambda, \phi \right)=\left\{\begin{array}{cc}\lambda & \mathrm{if}\ t>{t}_0\ \mathrm{and}\ \left(t \operatorname {mod}\ \phi =0\right)\\ {}0& \mathrm{else}\end{array}\right. $$

We also conservatively assumed, as in [25], that the senolytic drug kills only a susceptible subpopulation of the senescent cells, denoted *X*_SENSITIVE_, and does not affect the other senescent cells *X*_INSENSITIVE_. The total level of senescent cells is *X* = *X*_SENSITIVE_ + *X*_INSENSITIVE_.We denote by *ζ* the fraction of senescent cell production and noise that goes to make *X*_SENSITIVE_. The SR model dynamics with senolytics are therefore:

$$ {\dot{X}}_{\mathrm{SENSITIVE}}=\zeta \eta t-{\delta}_{t_0}\left(t,\lambda, \phi \right)X-\frac{\beta {X}_{\mathrm{SENSITIVE}}}{X+\kappa }+\sqrt{2\zeta \epsilon}\xi {\prime}_t $$

$$ {\dot{X}}_{\mathrm{INSENSITIVE}}=\left(1-\zeta \right)\eta t-\frac{\beta {X}_{\mathrm{INSENSITIVE}}}{X+\kappa }+\sqrt{2\left(1-\zeta \right)\epsilon}\xi^{\prime }{\prime}_t $$

For the simulations in Fig. 6, we used the human parameters from [25]: *η* = 0.00135 day^−1^year^−1^, *β* = 0.15 day^−1^, *κ* = 0.5, *ϵ* = 0.142 day^−1^, *X*_C_ = 17, as well as *ζ* = 0.25. In panel A, we used simulated senolytic dosage that corresponds to a strong removal of the sensitive senescent cells, *λ* = 0.5 day^−1^ given every *ϕ* = 60 days.
